# Four papers on child growth modelling

**DOI:** 10.1002/sim.8180

**Published:** 2019-06-11

**Authors:** Louise Ryan

**Affiliations:** ^1^ University of Technology Sydney Ultimo Australia; ^2^ Australian Research Council Centre of Excellence in Mathematical and Statistical Frontiers Parkville Australia

An initiative of the Bill & Melinda Gates Foundation, the knowledge integration project (http://kiglobalhealth.org) aims to improve global child health by promoting and supporting knowledge sharing among researchers. While the project has much broader potential, it began with and still has a primary focus on child growth and development. Such a focus is well warranted, since even with all today's modern advances in science and health, approximately a quarter of the world's children suffer from impaired growth due to nutritional deficiencies and other environmental factors.^1^ The project has created and continues adding to an impressive repository of data from studies conducted all over the world. Using this rich resource, researchers can gain a clearer picture of global trends and the factors that affect child birth and subsequent development, as well as improve their understanding of global variability in child health outcomes. Many of the studies in the repository have a longitudinal design, with children assessed repeatedly from birth and, in a couple of cases, even during the prenatal period. Physical growth, reflected through anthropomorphic measurements such as length or height, weight, and head circumference, is seen as a key factor in large part because it is well known that children with poor growth patterns early in life are at risk of a range of adverse health conditions in later childhood and adulthood.^2^, ^3^ Much of the early work in the project focused on the nontrivial task of amassing, organising, annotating, and making available the complex suite of data assets that had been contributed. Many conversations were held with various study investigators, hashing out important issues regarding protection of privacy and confidentiality, as well as policies around coauthorship. Given the size and complexity of the data, it was challenging for researchers to easily get a clear sense of what was available in the repository. This led to the development of a variety of innovative tools for exploring the project metadata and visualising the actual data (see http://kiglobalhealth.org/tools/). Over time, the strategy for engaging the many involved researchers also evolved and these days involves the use of “data science rallies” that draw on the principles of agile software development, bringing together a team to tackle a specific question with intense effort over a short period of time. While not necessarily “big data” in the sense of pure volume, modern data science tools and ways of thinking are a necessity when it comes to accessing the complex and diverse data available in the repository. It is no surprise that the project has unearthed a wealth of very interesting statistical challenges, particularly related to the analysis of longitudinal growth data in young children; hence, it was decided to bundle together several papers on the topic in this special issue.

The first two special issue papers by Ohuma and Altman^4^, ^5^ address the question of how to best construct age‐ and gender‐adjusted reference growth charts. Such charts are the basis through which parents might learn from the paediatrician that their child is, for example, in the 95% for length (or height for older children) and 90% for weight. Use of such standardised scores makes it much easier to monitor a child's growth trajectory over time. The first paper by Ohuma and Altman^4^ reviews the approaches that have routinely been used, based on cross‐sectional data, to construct reference growth charts and then propose some extensions to the longitudinal setting. As illustration, they use data from the INTERGROWTH‐21 project,^6^ which also includes prenatal head circumference data. The second paper by Ohuma and Altman^5^ draws from their experience with the INTERGROWTH‐21 study to offer design guidelines. The third and fourth papers in the special issue address the question of how to model repeated standardised growth measures in a longitudinal setting.^7^, ^8^ Anderson et al^7^ discuss the challenge of how to decide when there is evidence that a child has faltered in their growth, based on two successive standardised scores. In order not to be biased by regression to the mean, the difference between the successive scores needs to be adjusted by a term that reflects the correlation between the two measurements. Building on principles of meta‐analysis, the authors build a correlation matrix based on data from sixteen different studies from the repository. Anderson et al^8^ tackle the related question of how to best model a child's growth trajectory. They find that statistical models based on standardised scores (as opposed to length or height in centimetres or inches and weight in kilograms or pounds) tend to be easier to fit and less prone to model misspecification because they do not need to include potentially complex age and gender terms. Tim Cole, one of the world's leading experts in the analysis and modelling of child growth data, provides some interesting and provocative commentary on all four papers.^9^
